# 

**Published:** 1899-06

**Authors:** 


### NOTICES.

### Tennessee Dental Association.

The thirty-second annual meeting of the Tennessee Dental Association, will be held on Lookout Mountain, Chattanooga, July 4-6, 1899. An interesting program has been arranged and a cordial invitation is extended to all members of the profession.

N. C. LEONARD, *President.*
A. SIDNEY PAGE, *Secretary.*

### The Sixteenth Annual Session of the National Association of Dental Examiners.

The sixteenth annual session of the National Association of Dental Examiners will be held at Niagara Falls commencing at 10 o'clock, A. M., Friday, July 28th, and continuing the 29th, 31st and August 1st, 1899. The International Hotel will be the headqnarters and the rates will be $3.00, $3.50 and 4.00 per day according to location of rooms. As the sessions commence in advance of the National Association, write ahead and secure rooms as members of the N. A. of D. E. It is hoped that every

State will be represented by delegates with certificates signed by the President and Secretary of their respective boards.

CHARLES A. MEEKER, D.D.S., *Secretary.*
29 Fulton Street, Newark, N. J.

### The Thirty-fifth Annual Meeting Missouri State Dental Association.

The thirty-fifth annual meeting Missouri State Dental Association will be held in Kansas City, Mo., July 11, 12, 13 and 14, 1899. An interesting program will be presented.

Hotel and railroad rates have been secured.

All members of the profession are cordially invited to attend.

B. L. THORPE, *Cor. Secy.*
ST. LOUIS, MO.

### Michigan State Dental Society.

The annual meeting of this body will be held in Port Huron July, from the 11th to the 13th inclusive, 1899. A cordial and urgent invitation is extended to all members to be present, and take part in the proceedings, either by reading a paper, taking part in the discussions or by presenting some interesting and instructive case or cases, and describing any new methods or practice, or presenting any new specimens of any sort. Preparation has been made for an interesting meeting. This invitation is cordially extended to all legitimate members of the profession whether members of the society or not, and it is hoped that a large number of such will be present.

### National Dental Association.

The annual meeting of this body takes place at Niagara Falls on Tuesday, August 1, 1899. This is the second meeting under the new organization, and it is to be hoped that there will be renewed interest, energy and enthusiasm in carrying forward its legitimate work.

Indications are that there will be a large attendance. A program is to be issued in the near future, which we are assured will be one of great interest. Provision has already been made for the accomodation of the meeting at the hotels, and for a hall for the session. We are, judging from indications, fully warranted in saying that the sections are alive to their work.

Railroad rates have been secured from any and all points for one and one-third fare. Certificates being taken by the purchaser from the ticket agent, which will state the facts of the arrangement and entitle the holder to return to his home at one-third fare. We trust there will be a large attendance of the membership and a large number of visitors.

### American Medical Association—Section on Stomatology.

The complete program of this Section for the Columbus meeting of the Association is as follows:

1. Chairman's Address. G. V. I. Brown, Milwaukee.

2. The Human Face and Jaws as a Danger Signal of Systemic Defect or Disorder. J. G. Kiernan, Chicago.

3. Cocain and Eucain, their Relative Toxicity. A. H. Peck, Chicago.

4. Epithelial Structures in the Peridental Membrane. Frederick Noyes, Chicago.

5. Infectious Ulcerative Stomatitis. John S. Marshall, Chicago.

6. Oral Surgical Operation (with illustrations showing remarkable results). G. V. I. Brown, Milwaukee.

7. Some Points on Etiology, Pathology and Treatment of Persistent Pyorrhea Alveolaris. G. T. Carpenter, Chicago.

8. Interstitial Gingivitis (so-called Pyorrhea Alveolaris), giving the result of original work, with large microphotographic illustrations showing the progress of the disease from the beginning to the Exfoliation of the Teeth. Eugene S. Talbot, Chicago.

9. Syphilitic Infection from Dental Instruments, with Cases. W. L. Baum, Chicago.

10. Therapeutics of Inflammation. W. B. Hill, Millwaukee.

11. Professional Education and Ethics. E. E. Baldwin, Chicago.

12. Neuralgias Due to Progressive Lesions of the Periosteum. M. H. Fletcher, Cincinnati.

13. Treatment and Positive Cure of Pyorrhea Alveolaris in

Connection with Restoration of Normal Articulation. W. G. A. Bonwill, Philadelphia.

14. So-called Pyorrhea Alveolaris. Euphraim Cutter, New York City.

Dr. Bonwill will hold clinics independent of the meetings of the Section, to those who wish to meet him.

